# Plasma d-dimer level correlated with advanced breast carcinoma in female patients

**DOI:** 10.1016/j.amsu.2018.10.025

**Published:** 2018-10-26

**Authors:** Basim Rassam Ghadhban

**Affiliations:** College of Medicine, Baghdad University, Baghdad Teaching Hospital/ Department of Surgery, Iraq

**Keywords:** D-dimer level, Hyper coagulation, Advanced breast cancer, Metastasis

## Abstract

**Background:**

Advanced breast cancer is a common disease among female in the world. There is a correlation between cancer and hyper coagulation. In a cancer state, there is an increase in the level of cross-linked fibrin degradation product (d-dimer)which indicates systemic activation of fibrinolysis and hemostasis. So, there is a relation between increase d-dimer value and advanced breast disease.

**Objective:**

To confirm the relation between increase d-dimer levels and advanced breast carcinoma in female patients.

**Patients and methods:**

A prospective study (cohort study) done in Baghdad teaching hospital (department of surgery) from Jan 2014 to Jan 2016. Seventy patients were categorized intotwo equal groups, group 1 with breast carcinoma, group 2 with benign breast tumor. Plasma d-dimer levels compared for each group, and in relation to (tumor size, stage, grade, lympho-vascular invasion, and lymph nodes involvement).

**Results:**

D-dimer level was normal in group two (<0.25) mg/l and high in group one in other words, d-dimer level was increasing in advanced breast carcinoma group with enlarge tumor size, high stage, grade, lympho-vascular invasion and lymph nodes involvement.

**Conclusions:**

Plasma d-dimer levels was good prognostic factor in breast carcinoma specially in advanced breast carcinoma and its considered factor clinical stage progression lympho-vascular invasion and metastasis.

## 1 Introduction

Breast cancer in female is the most common malignant neoplasm and represents a diversified group of tumors, which exhibit different behaviors and altered response to therapy. Biological markers, hormonal status, histological grading and subgroups status, tumor size, lymph node embroilment have predictive and/or prognostic value and they are the important factors in nominate appropriate treatments [1].

Although clinical and experimental trials have demonstrated the relationship between cancer and hemostasis but the exact mechanism is not fully understood [2].

Thus, systemic activation of coagulation and hemostatic system in all cancer patients without thromboembolism have been still under investigation [2].

Advanced breast cancer is either locally advanced or metastatic spread. There is correlation between cancer and hypercoagulation, global hemostasis is more frequently activated in patients with cancer. This systemic activation has been included in (angiogenesis, progression, metastatic spread) of tumor cells. Elevated levels of d-dimer, which is produced by degradation of cross-linked fibrin; indicate global activation of fibrinolysis and hemostasis [3].

In breast cancer, an elevation of plasma d-dimer is linked and correlated with locally advanced breast cancer or metastasis to axillary lymph nodes or distant metastasis, advanced breast cancer includes the most serious of the five possible stages (stages 3 & 4) [4].

Stage 3 is locally advanced breast carcinoma, in other words, the disease has metastasis to lymph nodes or another tissue in the breast but not to farther sites in the body, while stage 4 of the disease metastatic breast cancer to other organs mainly the liver, lungs, bones, brain [5].

The foremost step in tumor metastasis is remodeling and fibrin deposition in the tumor extracellular matrix. A tumor to be successfully metastasize from its original site, it must undergo many coerce steps, this including the invasion into either the vascular or lymphatic lumen, conveyance through the circulation, and establishment of viability in base tissues. Cross-linked fibrin serves as a stable framework in the extracellular matrix for endothelial cell migration when tumor cell migration and angiogenesis while invasion [6].

Remodeling of extracellular fibrin is primary for angiogenesis in tumors, and activation of intravascular fibrin fashioning and dissolution is occurring in the plasma of the patients. In apposition to other indices of fibrinolytic pathway activation, like levels of plasminogen activator inhibitor and prolinase plasminogen activator, which shown prognostic significance in breast cancer's patients [7].

Furthermore, activation of coagulation system, minutely thrombin generation and fibrin figuration and degradation, have been included in angiogenesis, tumor progression, tumor cell stealth and metastatic spread [8]. Thrombin is a fundamental enzyme in the process of blood coagulation and leads to the transformation of fibrinogen to fibrin, which is the end result of blood coagulation and lastly gives rise to the formation of a fibrin clot. Tumor cells also retain intensive procoagulant activities that stimulate regional activation of the coagulation system and deposition of fibrin.

The aim of this study is to confirm the relation between increase d-dimer levels and advanced breast carcinoma in female patients.

**Ethical consideration:** this research is done under supervision of Ethical committee of department of surgery (Baghdad college of medicine).

**Statistical analysis:** statistical package for social sciences (SPSS) for Windows 10.0 program was used. for descriptive statistical mean, standard deviation, frequency, and median were used.

## 2 Materials and methods

This study was done at Baghdad Teaching Hospital (Department of Surgery) from Jan 2014 to Jan 2016. Seventy female patients with breast cancer were included in this study. They were divided into two groups according to history clinical examination and triple assessment of the disease. Group 1; included 35 patients diagnosed with malignant breast cancer their age ranged between 25 and 65 years. The other 35 patients (Group two) were diagnosed as having benign breast disease and their age ranged between 20 and 50 years.

**Exclusion criteria**: we excluded (11 patients):

1 patients with other cancer e.g. cervical and colorectal carcinoma (1 patient).
2 smokers (5 patients)
3 patients with venous thromboembolic diseases (2 patients)
4 unstable angina (1 patient)
5 severe infection (pneumonia) (1 patient)
6 patient on Aspirin (1 patient)

Blood venous samples (3 ml) were collected from all patients before any surgical intervention, and clinical staging was done including; tumor size, site, nodal involvement, distant metastases (TNM). Ultrasound of abdomen and chest x-ray were taken to each patient. Grading of the disease was done by histopathological study including lympho-involvement, lymphovascular invasion, and number of lymph nodes involved by tumor. The patients who were enrolled in the present study were treated by either lumpectomy or modified mastectomy and axillary dissection. The samples were sent for histopathological study as mentioned above. The data were analyzed by standard deviation and p value in comparison between two groups. P < 0.05 was considered significant [9].

## 3 Results

All patients were enrolled in our study were females. Seventy patients were randomly selected, age in group 1 ranged from 25 to 65 years (mean 45) while in group 2 ranged from 20 to 50 years old (mean 35). In 70% of the patients, the site of the lump was upper outer quadrant of the breast.

In group 1, 30% were premenopausal and 70% were postmenopausal while in group 2, 98% of them were premenopausal and 2% only were postmenopausal.

In group 1 four (11.4%) patients had d-dimer level<0.25 mg/l, 11 (31.4%) patients had level ranged (0.25–0.50 mg/l), 13 (37.1%) patients had level ranged (0.5–1 mg/l) and 7(20%) patients had level ranged (1–2 mg/l), while in group 2, 34 (97.1%) patients have level (0.25 mg/l) and only 1 patient (2.8%) had level ranged (0.25–0.50 mg/l) as shown in Table 1.

**Table 1:** Level of plasma d-dimer among group 1 and group 2 patients studied.

d-dimer level (mg/l)	Patients in group 1	Patients in group 2
0.25	4(11.4%)	34(97.1%)
(0.25–0.50)	11(31.4%)	1(2.8%)
(0.5–1)	13(37.1%)	–
[1,2]	7(20%)	–
TOTAL	35	35

Table 2 showed the mean and standard deviation of d-dimer level distributed according to stage of the disease. It was found that d-dimer level was significantly elevated in breast cancer patients compared with group 2. In patients with stageIIa and IIb showed significantly differences (0.4 ± 0.25820, 0.4857 ± 0.27946) compared with normal d-dimer level in group 2. Stage IIIa and stage IIIb showed highly significant differences (0.5200 ± 0.25884, 0.6455 ± 0.28413) in normal value of group 2.

**Table 2:** The mean, standard deviation (Mean ± SD) of d-dimer level distributed according to stage of the disease.

Stages of disease	Mean of d-dimer level^a^	No. of patients studied	Std. Deviation
Stage I	0.1000	2	0.00000
stage II a	0.4000	5	0.25820
stage II b	0.4857	8	0.27946
stage III a	0.5200	6	0.25884
stage III b	0.6455	12	0.28413
stage III c	1.1000	1	.
stage IV	1.0000	1	.

a Normal d-dimer level<0.25 mg/l.

It was noticed that when there was increase in tumor size there was also an elevation in mean of plasma d-dimer level. In T1, four patients with tumor size had d-dimer value 7.2 mg/l, 12 patients with tumor size T2 had mean d-dimer (29.17 mg/l), five patients with tumor size T3 had (8.9 mg/l) mean of the plasma d-dimer level, other 14 patients had tumor size T 4 with d-dimer level of 54.12 mg/l, (Table 3).

**Table 3:** The tumor size distributed according to the level of the plasma d-dimer in group one.

Tumor size type	Number of patients	Mean of D-dimer (mg/L)
T1 (<2 cm)	4	7.2400
T2 (2–5 cm)	12	29.1700
T3 (>5 cm)	5	8.9000
T4 (any size spread beyond breast tissue)	14	54.1700
Total	35	34.3347

A significant relationship was observed between histopathological grade and mean of level d-dimer at p value ≤ 0.01. The result revealed when there was an increase grade of the disease, there was elevation in the level of d-dimer. In group one,7 patients (20.0%), with histopathologically grade I tumor, had the mean of d-dimer (1.8 mg/l), while 12 patients (34.3%) with grade II disease, had mean of 2.8 mg/l. Other 16 patients (45.7%) with grade III had mean level of plasma d-dimer (12.4) mg/l. The difference was significant at p value ≥ 0.01, (Table 4).

**Table 4:** Histopathological grade distributed in comparison to the level of the plasma d-dimer.

Histopathological grade	No. of patients	d-dimer mean
I	7	1.7 (10.06)
II	12	2.8 (16.57)
III	16	12.4 (73.37)

Significant at p value ≤ 0.01.

Table 5 shows lympho-vascular invasion of the disease distributed according d-dimer level. In group A, out of 24 patients (68.6%) with lympho-vascular invasion had mean value of (4.2** **mg/l) while group B 11 patients (31.4%) had no lympho-vascular invasion with the mean of d-dimer with (1.7 mg/l), (Table 5).

**Table 5:** Lympho-vascular invasion of the disease distributed according d-dimer level.

Lymphovascular invasion of the disease	Number of patients (%)	Mean of d-dimer level (mg/l)
Group A (with lymphovascular invasion)	24(68.6%)	4.2
Group B (without lymphovascular invasion)	11(31.4%)	1.7
Total	35(100%)	

Table 6 showed lymph nodes involved among patient studied distributed according to d-dimer level. In group A out of 29 patients (82.9%) with lymph nodes involvement and the d-dimer level with mean of (4 mg/l), group B only 6 patients (17.1%) with no lymph node involvement had mean d-dimer level of (0.25 mg/l).

**Table 6:** Lymph nodes involved among patient studied distributed according to d-dimer level.

Lymph nodes involved	Number of patient (%)	Mean of d-dimer level(mg/l)
Group A (Involved)	29 (82.4)	4
Group B (Not involved)	6(17.6)	0.25
Total	35(100%)	

## 4 Discussion

The fibrin d-dimer antigen is unparalleled marker of the primary enzymatic dissolution product of cross-linked fibrin by plasmin dissolute the cross-linked fibrin to liberate fibrin degradation products and reveal the D dimer antigen. Systemic values of D-dimer are an index of fibrin transition in the circulation [9]. Plasma D dimer levels are elevated in many clinical conditions like smoking, infection, pregnancy, old age, trauma, tumors and others [11], addendum to the diagnostic use of D dimer, it could be of conceivable prognostic use in many conditions.

D-dimer increases in various disorders including venothromboembolic (VTE), cardiovascular disease and cancer. In addition, elevated d-dimer levels were shown in healthy adult population. Although the pathophysiology of this activation is not completely yet understood, studies have been reported that observed it in cancer patients without thromboembolism. Also, it has been shown in the present study that elevated d-dimer level had an important prognostic role on prognosis of the breast cancer, many studies documented the role of the plasma d-dimer who have prognostic value in many types of cancers. In a study performed by Nagy et al. and Dirix et al. [12,13] who was established a relationship between elevated d-dimer levels in patients with breast cancer and elevated tumor markers lead to increased mortality risks. This result was in agreement with the present study. In one study performed by Di Castelnuovo et al. [14]who noticed that elevated d-Dimer level was an independent of the presence of distant metastasis.

Tumor grade, nodal status and size stayed the most important prognostic factors for long-dated survival, though their role decreased over time [15]. Strong correlation were noticed between the lymph node involvement which is the important for the affirmation of clinical stage and treatment, clinical stage and number of metastatic nodules with plasm d-dimer levels. Our results were in agree with Blackwell et al. [16]. Also, documented that plasma d-dimer level was lymphovascular invasion marker, clinical stage, and lymph node involvement in operable breast cancer. Zhang et al. [17] also suggested that detectable fibrin dissolution, as measured by plasma D-dimer, is a clinically essential marker for lymphovascular invasion and going early tumor metastasis in operable breast cancer. Di Micco et al. [18]also proclaim that plasma d-dimer levels were increased in gastric cancer patients but the possible correlation between plasma d-dimer levels. The conclusion of the present study also specified that plasma d-dimer levels were elevated in patients with extensive tumors, advanced T, N and TNM stage in patients studied.

Joensuu et al. [19]examined renaissance rates among patients revealed by screening compared to those discovered outside screening. After modification for tumor aggressiveness (tumor grade, nodal status, size, age, treatment, PR status, HER-2), hence excluding bias towards detection of inactive cancers (length bias), the avail of screening for the prognosis for BC(breast cancer) patients stayed evident, this indicate that other agents explain the indolent demeanor of BC revealed by screening. Hence, until this agent is established, discovery mode should probably be counted as a prognostic factor and thus be considered into account in patient management [20].

## 5 Conclusions

Plasma d-dimer levels was good prognostic factor in breast carcinoma especially in advanced breast carcinoma and may also consider a good indicator for determining clinical stage progression of the disease, lympho-vascular invasion and metastasis. Activation coagulation and fibrinolysis system in patients with cancer is combined with tumor stoma formation and metastasis in various cancer types.

## Recommendation

After time linear studies with sequent measurements of d-dimer levels would be requisite to analyze their association with disease progression advanced breast cancer we also recommend using of INR, PT in addition to d-dimer value. We noticed that INR, PT also were increased in same time with the increase of d-dimer level in relation to increase (tumor size, stage of disease, lymph node involvement, and lympho-vascular invasion).

## Ethical Approval

Yes **Ethical Approval** and patient consent under supervision of ethical committee of surgery Department of Surgery, Baghdad University, Medical City.

## Sources of funding

There is no funding for my research.

## Author contribution

Submitted by Dr Basim Rassam Ghadhban.

And with the help of Baghdad Teaching Hospital/Department of surgery.

## Conflicts of interest

There is no conflicts of interest.

## Research Registration number

108678.

## Guarantor

Dr Basim Rassam Ghadhban.

Mail:basimgadban@yahoo.com.

## Provenance and peer review

Not commissioned, externally peer reviewed.

## Appendix A Supplementary data

Process Checklist Process Checklist
