# Supplementary material for: Plasma d-dimer level correlated with advanced breast carcinoma in female patients
Source: Ann Med Surg (Lond). 2018 Oct 26;36:75–8. doi: 10.1016/j.amsu.2018.10.025 (PMC6215958; doi:10.1016/j.amsu.2018.10.025)
Supplement: Process Checklist [file mmc1.docx]

| PROCESS CHECKLIST | | | |
| --- | --- | --- | --- |
| Section | **Item** | **Checklist Description** | **Page Number** |
| Title | 1 | **Plasma d-dimer Level Correlated with Advanced Breast Carcinoma in Female Patients**  -case series |  |
| Abstract | 2a | **Abstract**  **Background:** Advanced breast cancer is a common disease among female in the world.There is a correlation between cancer and hyper coagulation. In a cancer state, there is an increase in the level of cross-linked fibrin degradation product (d-dimer)which indicates systemic activation of fibrinolysis and hemostasis. So, there is a relation between increase d-dimer value and advanced breast disease. |  |
|  | 2b | **Patients and methods:** A prospective study (cohort study) done in Baghdad teaching hospital (department of surgery) from Jan 2014 to Jan 2016.Seventy patients were categorized intotwo equal groups, group 1 with breast carcinoma, group 2 with benign breast tumor.Plasma d-dimer levels compared for each group, and in relation to (tumor size, stage, grade, lympho-vascular invasion, and lymph nodes involvement).  . |  |
|  | 2c | **Results:** D-dimer level was normal in group two (<0.25) mg/l and high in group one in other words, d-dimer level was increasing in advanced breast carcinoma group with enlarge tumor size, high stage, grade, lympho-vascular invasion and lymph nodes involvement. |  |
|  | 2d | **Conclusions:** Plasma d-dimer levels was good prognostic factor in breast carcinoma specially in advanced breast carcinoma and its considered factor clinical stage progression lympho-vascular invasion and metastasis. |  |
| Introduction | 3 | **Introduction**  Breast cancer in female is the most common malignant neoplasm and represents a diversified group of tumors, which exhibit different behaviors and altered response to therapy. Biological markers, hormonal status, histological grading and subgroups status, tumor size, lymph node embroilment have predictive and/or prognostic value and they are the important factors in nominate appropriate treatments ^(1)^.  Although clinical and experimental trials have demonstrated the relationship between cancer and hemostasis but the exact mechanism is not fully understood ^(2)^.  Thus, systemic activation of coagulation and hemostatic system in all cancer patients without thromboembolism have been still under investigation^(2)^.  Advanced breast cancer is either locally advanced or metastatic spread. There is correlation between cancer and hypercoagulation, global hemostasis is more frequently activated in patients with cancer. This systemic activation has been included in (angiogenesis, progression, metastatic spread) of tumor cells. Elevated levels of d-dimer, which is produced by degradation of cross-linked fibrin; indicate global activation of fibrinolysis and hemostasis^(3)^.  In breast cancer, an elevation of plasma d-dimer is linked and correlated with locally advanced breast cancer or metastasis to axillary lymph nodes or distant metastasis, advanced breast cancer includes the most serious of the five possible stages (stages 3 & 4)^(4)^.  Stage 3 is locally advanced breast carcinoma, in other words, the disease has metastasis to lymph nodes or another tissue in the breast but not to farther sites in the body, while stage 4  of the disease metastatic breast cancer to other organs mainly the liver, lungs, bones, brain^(5)^.  The foremost step in tumor metastasis is remodeling and fibrin deposition in the tumor extracellular matrix. A tumor to be successfully metastasize from its original site, it must undergo many coerce steps, this including the invasion into either the vascular or lymphatic lumen, conveyance through the circulation, and establishment of viability in base tissues. Cross-linked fibrin serves as a stable framework in the extracellular matrix for endothelial cell migration when tumor cell migration and angiogenesis while invasion^(6)^.  Remodeling of extracellular fibrin is primary for angiogenesis in tumors, and activation of intravascular fibrin fashioning and dissolution is occurring in the plasma of the patients. In apposition to other indices of fibrinolytic pathway activation, like levels of plasminogen activator inhibitor and prolinase plasminogen activator, which shown prognostic significance in breast cancer’s patients.^(7)^.  Furthermore, activation of coagulation system, minutely thrombin generation and fibrin figuration and degradation, have been included in angiogenesis, tumor progression, tumor cell stealth and metastatic spread^(8)^.Thrombin is a fundamental enzyme in the process of blood coagulation and leads to the transformation of fibrinogen to fibrin, which is the end result of blood coagulation and lastly gives rise to the formation of a fibrin clot. Tumor cells also retain intensive procoagulant activities that stimulate regional activation of the coagulation system and deposition of fibrin.  The aim of this study is to confirm the relation between increase d-dimer levels and advanced breast carcinoma in female patients. |  |
| Methods | 4a | **Materials and Methods:**  This study was done at Baghdad Teaching Hospital (Department of Surgery) from Jan 2014 to Jan 2016.Seventy female patients with breast cancer were included in this study. They were divided into two groups according to history clinical examination and triple assessment of the disease. Group 1 ; included 35 patients diagnosed with malignant breast cancer their age ranged between 25 and 65 years. The other 35 patients (Group two) were diagnosed as having benign breast disease and their age ranged between 20 and 50 years.  **Exclusion criteria**: we excluded (11 patients) :  1-patients with other cancer e.g. cervical and colorectal carcinoma (1 patient).  2-smokers (5 patients)  3- patients with venous thromboembolic diseases (2 patients)  4- unstable angina (1 patient)  5-severe infection (pneumonia) (1 patient)  6-patient on Aspirin (1 patient)  Blood venous samples (3 ml) were collected from all patients before any surgical intervention, and clinical staging was done including; tumor size, site, nodal involvement, distant metastases (TNM). Ultrasound of abdomen and chest x-ray were taken to each patient. Grading of the disease was done by histopathological study including lympho-involvement, lymphovascular invasion, and number of lymph nodes involved by tumor. The patients who were enrolled in the present study were treated by either lumpectomy or modified mastectomy and axillary dissection. The samples were sent for histopathological study as mentioned above. The data were analyzed by standard deviation and p value in comparison between two groups. P<0.05 was considered significant ^(9)^. |  |
|  | 4b | **Study design** |  |
|  | 4c | **Setting**  Describe the setting(s)and nature of the institution in which the patient was managed; academic, community or private practice setting? Location(s), and relevant dates, including periods of recruitment, exposure, follow-up, and data collection |  |
|  | 4d | **Participants**  Describe the relevant characteristics of the participants (comorbidities, tumour staging, smoking status, etc). State any eligibility (inclusion/exclusion) criteria and the sources and methods of selection of participants. Describe length and methods of follow-up. |  |
|  | 4e | **Pre-intervention considerations**  e.g. Patient optimisation: measures taken prior to surgery or other intervention e.g. treating hypothermia/hypovolaemia/hypotension in burns patients, ICU care for sepsis, dealing with anticoagulation/other medications and so on. |  |
|  | 4f | **Types of intervention(s) deployed**  To include reasoning behind treatment offered (pharmacological, surgical, physiotherapy, psychological, preventive) and concurrent treatments (antibiotics, analgesia, anti-emetics, nil by mouth, VTE prophylaxis, etc). Medical devices should have manufacturer and model specifically mentioned. |  |
|  | 4g | **Peri-intervention considerations**  Administration of intervention (what, where, when and how was it done, including details for surgery; anaesthesia, patient position, use of tourniquet and other relevant equipment, preparation used, sutures, devices, surgical stage (1 or 2 stage, etc) and operative time. Pharmacological therapies should include formulation, dosage, strength, route and duration). Authors are encouraged to use figures, diagrams, photos, video and other multimedia to explain their intervention. |  |
|  | 4h | **Who performed the procedure(s)**  Operator experience (position on the learning curve for the technique if established, specialisation and prior relevant training). |  |
|  | 4i | **Quality control**  What measures were taken to reduce inter or intra-operator variation. What measures were taken to ensure quality and consistency in the delivery of the intervention e.g. independent observers, lymph node counts, etc |  |
|  | 4j | **Post-intervention considerations**  e.g. post-operative instructions and place of care. Important follow-up measures - diagnostic and other test results. Future surveillance requirements - e.g. imaging surveillance of endovascular aneurysm repair (EVAR) or clinical exam/ultrasound of regional lymph nodes for skin cancer. |  |
| Results | 5a | **Participants**  Report numbers involved and their characteristics (co-morbidities, tumour staging, smoking status, etc). |  |
|  | 5b | **Changes**  Any changes in the interventions during the course of the case series (how has it evolved, been altered or tinkered with, what learning occurred, etc) together with rationale and a diagram if appropriate. Degree of novelty for a surgical technique/device should be mentioned and a comment on learning curves should be made for new techniques/devices. |  |
|  | 5c | **Outcomes and follow-up**  Clinician assessed and patient-reported outcomes (when appropriate) should be stated with inclusion of the time periods at which assessed. Relevant photographs/radiological images should be provided e.g. 12-month follow-up. |  |
|  | 5d | **Intervention adherence/compliance and tolerability**  How was this assessed. Describe loss to follow-up (express as a percentage and a fraction) and any explanations for it. |  |
|  | 5e | **Complications and adverse or unanticipated events**  Described in detail and ideally categorised in accordance with the Clavien-Dindo Classification. How they were prevented, mitigated, diagnosed and managed. Blood loss, wound complications, re-exploration/revision surgery, 30-day post-op and long-term morbidity/mortality may need to be specified. |  |
| Discussion | 6a | **Summarise key results** |  |
|  | 6b | **Discussion of relevance**  Relevant literature, implications for clinical practice guidelines, how have the indications for a new technique/device been refined and how do outcomes compare with established therapies and the prevailing gold standard should one exist and any relevant hypothesis generation. |  |
|  | 6c | **Strengths and limitations of the study** |  |
|  | 6d | **The rationale for any conclusions?** |  |
| Conclusions | 7a | **State the key conclusions from the study** |  |
|  | 7b | **State what needs to be done next, further research with what study design.** |  |
| Additional Information | 8a | **State any conflicts of interest** |  |
|  | 8b | **State any sources of funding** |  |
